# Transitioning to midwifery models of care: lessons from case studies in India

**DOI:** 10.1080/16549716.2025.2522502

**Published:** 2025-09-17

**Authors:** Paridhi Jha, Bharati Sharma, Alison McFadden, Sowmya Thota, Joyce Marshall, Neha Singh, Sowmya Ramesh, Jitender Nagpal, Andrew Symon

**Affiliations:** a Foundation for Research in Health Systems, Indian Society for Health Administrators Building, Bengaluru, India; b Indian Institute of Public Health, Gandhinagar, Gujarat, India; c Mother and Infant Research Unit, School of Health Sciences, University of Dundee, Dundee, UK; d Fernandez Hospital Educational and Research Foundation, Hyderabad, Telangana, India; e University of Huddersfield, Huddersfield, UK; f Quicksand Design Studio Pvt. Ltd, New Delhi, India; g North Bristol NHS Trust, Department of Infection, Bristol, UK; h Sitaram Bhartia Institute of Science and Research, New Delhi, India

**Keywords:** Midwife-led care units, health systems research, quality of maternity services, QMNC framework, ecological systems theory, framework analysis

## Abstract

**Background:**

Limited evidence exists on how midwives, other healthcare providers, and their local communities in low- and middle-income countries experience the transition to a midwifery model of care.

**Objectives:**

This study aimed to identify opportunities, enablers, and barriers to integrating midwifery into existing public health systems, from the perspectives of key stakeholders representing health systems and communities.

**Method:**

We conducted two case studies – community-based and public health facility-based – in Gujarat and Telangana, comprising 63 in-depth interviews and five focus group discussions with 40 participants, including midwives, obstetricians, nurse-midwives, hospital administrators, local women, family members, and community influencers. The ecological systems theory and the quality maternal and newborn care framework guided the framework analysis.

**Results:**

Five themes were developed, each aligning with one level of ecological systems theory: 1) Becoming a midwife; 2) Negotiating the full scope of autonomous midwifery practice; 3) Midwives’ integration into existing maternity care teams; 4) Building social recognition of midwifery in communities; and 5) Balancing the care context and policy directives. Midwives had to demonstrate professional competence, confidence, and assertiveness to overcome professional challenges and conflicts and gain acceptance.

**Conclusion:**

Integrating midwifery into existing health systems depends on midwives’ acceptance and perceived trustworthiness by existing health teams/communities and can be facilitated by ongoing support and mentoring of midwives, preparatory sensitisation of other healthcare providers, and building social recognition of midwives in communities. Further research is needed to develop and test interventions that support midwives in fulfilling their potential to improve the care quality for mothers and infants.

## Paper Context

- **Main findings:** Indian professional midwives need mentorship and coordinated policy-level support to facilitate integration into existing health teams. Conflicts seem common in India between midwives and other maternity care providers, rooted in professional competition, pay disparities, and role insecurities. Sensitizing medical officers and hospital superintendents and raising awareness in communities uniformly will help midwives integrate smoothly into existing health teams in India.
- **Added knowledge:** The findings add novel insights into integrating midwifery into a health system transitioning to a midwife-led model of care.
- **Global health impact for policy and action:** Given the global movement towards transitioning to midwife-led models of care, the findings are relevant to low – and middle-income countries that are currently in transition.

## Background

Global evidence estimates that maternal and perinatal deaths worldwide could be reduced by 67% and 64%, respectively, by implementing the complete package of midwifery care, including family planning and maternal and neonatal health interventions [1]. In addition, over 50 clinical and other outcomes are known to be improved by high-quality midwifery care, including reduced preterm birth, reduced interventions, and enhanced maternal satisfaction and well-being [2]. Midwives educated to international standards can provide 87% of maternity services [3].

In recognition of the impact midwives can have, the World Health Organization published a position paper for low- and middle-income countries (LMICs) without a strong cadre of professional midwives to transition to midwifery models of care [4]. The paper recognises that midwifery models of care are cost-effective and improve outcomes for women and newborns. The paper also highlights the importance of the integration of midwives within interdisciplinary teams [4]. The WHO paper highlights the need for implementation research on transitioning to midwifery models of care in LMICS. To contribute to this evidence base, we conducted two case studies examining the barriers and facilitators to integrating midwifery in India. We define integration as the process of deploying newly educated midwives as a distinct profession that is accepted and respected by the multi-disciplinary team, into existing health systems. This is an important step in transitioning to a fully operational midwife-led model of care that can be scaled-up across India.

India accounts for 20% of births globally [5] and has high – albeit declining – rates of maternal and perinatal mortality (MMR 97 per 100,000 births; PMR 23 per 1000 live births [6]. The Indian government aims to reduce the MMR to under 70 and the PMR to less than 12 by 2030, reflecting the WHO’s Sustainable Development Goals (SDGs). The Government of India launched India’s first midwifery service guidelines in 2018 [7] as a strategy to improve the quality of maternity care and reduce persistently poor birth outcomes. There is a policy-level commitment at both the national and state levels to promote respectful care and to stop non-evidence-based practices [8]. These include high and increasing rates of unnecessary interventions, especially caesarean sections.

## Midwifery education and services in India

Since independence in 1947, midwifery education in India has been subsumed within two distinct nursing programmes: the 3.5-year General Nursing and Midwifery (GNM) diploma course and a 4.5-year bachelor’s degree in nursing. Both are regulated nationally by the Indian Nursing Council and at the state level by State Nursing and Midwifery Councils [9]. Graduating students are awarded dual licensure as Registered Nurses (RNs) and Registered Midwives (RMs). They can work as staff nurse-midwives (SNMs) in Indian public and private health service sectors. Two additional workforce groups are involved in and are directly relevant to maternity care in India. Auxiliary Nurses and Midwives (ANMs) are women who have undertaken a 24-month course on basic nursing and midwifery skills [10] and have received a Registered ANM (RANM) registration from the Indian and State Nursing Councils. ANMs are attached to a public health facility in their community, providing community-based pre-pregnancy, antenatal, postnatal, and family planning services to women and families [11]. They may provide intrapartum care at the local facility if staffing levels require this [12]. Accredited Social Health Activists (ASHAs) are female community-level change agents and advocates who empower pregnant women in communities through education, information, and general assistance to attend health facilities for childbirth [13].

However, none of these programmes meet the global standards for educating professional midwives [14]. The Indian Government’s 2018 National Midwifery Guidelines aimed to establish National and State-level Midwifery Training Institutes (NMTIs; SMTIs) by investing in training and mentoring a group of midwifery educators before rolling out an 18-month midwifery education programme for eligible nurse-midwives [7] to prepare professional midwives who are educated as per the global standards of midwifery education. The initiative aims to deploy the graduates from this education programme as Nurse Practitioners in Midwifery (NPMs), a new cadre of professional midwives in India’s public health system [7].

Studies report that the Government of India’s policy of deploying NPMs faces some challenges [9,15,16]. Barriers include a lack of knowledge and skills among birth attendants [9], skill loss among midwives due to not practising to their full potential, and limited career development opportunities [14,15]. However, these studies provide limited insights into the perceptions, experiences, facilitators, and barriers associated with integrating professional midwives into existing health systems. Consequently, it is challenging to identify potential areas for improvement.

Our study aimed to i) assess stakeholder perceptions of the quality of current maternity services and the acceptability of midwives as key providers of maternal and neonatal care and ii) identify stakeholders’ perceptions of the barriers and facilitators to integrating professional midwives into current maternity services. We selected the states of Gujarat and Telangana as the study settings because they had already deployed NPMs in public health facilities when our study commenced. Since the conclusion of this study in 2023, midwifery educators’ training and deployment has started in several other states.

### Professional midwifery education in Gujarat and Telangana states

Table 1 presents a comparison of the two states’ different approaches to introducing professional midwifery into their public health and education systems:

**Table 1.** Comparison of the professional midwifery education and services in Gujarat and Telangana.

Indicators	Gujarat	Telangana
Initiation of preparing NPMs	2009–2017 (thereafter, the state has adopted the course in line with GoI guidelines) [7]	In 2011. The state adopted the private-sector model in 2015, later adapted as the national model in 2018.
Course Curriculum	Implemented the Indian Nursing Council’s old 11-month NPM course	An 18-month midwifery education programme [17] followed by a six-month supervised internship. The Albany Midwifery Practice in UK was chosen as an index model due to the former’s success in making services accessible to those from deprived/excluded communities [17]
NPM course sites	Six public sector colleges of nursing	The private-sector Fernandez Hospital group first developed and implemented the programme in their hospital, which was later adopted by the State Government at two public-sector colleges of nursing with support from UNICEF [17]. The model was adopted by National Government [7]
Competence of educators	Postgraduate or PhD degree in Gynaecological Nursing and Midwifery Experienced in teaching midwifery to students of integrated courses (GNM/BSc Nursing) May not have had recent clinical experience in midwifery [18].	International midwifery educators meeting global midwifery education standards [14] and core competencies [19] were recruited from the UK [17]. Gradually prepared native midwifery educators from Telangana.
Clinical education of NPMs	Under the tutelage of obstetricians	Supervised by international midwifery educators
NPMs educated	About 450 NPMs were educated between 2009 and 2017 before the 2018 National Guidelines were launched.	About 30 were educated and absorbed in Fernandez Group’s private-sector model. Up to 2018, 45 midwives had qualified and were deployed in public health facilities in Telangana.
Current state policy	Since 2018, the Gujarat model of midwifery education has been adapted in line with the 2018 National Guidelines. State policy is first to develop a bridging course to align the original 450 NPMs with the new 18-month professional midwifery education programme. Subsequently, all new enrolments will undertake the new 18-month programme.	The training of NPMs continues in line with the 2018 guidelines [7]

### Context of this study

Public health facilities hosting the specially created ‘Midwife-led Care Units’ (MLCUs) were selected for this study as government policies prioritise training and the deployment of NPMs within the public sector. The midwives’ deployment started from higher- to lower-level health facilities. Table 2 presents a brief description of different levels of Indian public health facilities.

**Table 2.** Levels of health facilities in the Indian public health system relevant to our study.

Health Facility	Catering to	Services Provided	Maternity Care Team Composition
Medical College Hospital (MCH)	Typically, 500+ bedded hospitals. Community within 500 square kilometres area, referral point for all district hospitals in this area	Super-specialty services, including Emergency Obstetric Care, infertility treatment, caesarean births, neonatal complications and intensive care. May cater to 8000–12000 annual births, may exceed	Several experienced obstetricians/paediatricians are supported by their teams of resident doctors, masters’ and graduate-level medical students. All are supported by the SNMs working in maternity wards. Supports clinical learning of physicians, nurses and midwives
District Hospitals (DH)	100–500 bedded multi-speciality hospital catering to 300,000–400,000 population [20]	Some speciality services, including Emergency Obstetric Care, caesarean births, neonatal complications and intensive care. May cater to 8000–12000 annual births, may exceed	4–6 obstetricians, 4–6 Paediatricians supported by a team of SNMs May or may not be a clinical learning site for medical/nursing students
Area Hospitals (AH)/Sub District Hospitals (SDH)	80–200 bedded hospitals in larger Indian districts (population ≥ 5 million) to support the District Hospital in providing services	Some speciality services, including Emergency Obstetric Care, caesarean births, neonatal complications and intensive care. May cater to 8000–12000 annual births, may exceed	4–6 obstetricians, 4–6 Paediatricians supported by a team of SNMs It may or may not be a clinical learning site for medical/nursing students
Community Health Centers (CHCs)	30–60 bedded first referral units catering to 120,000–150,000 population [21]	First referral Unit of Indian Public Health System. Each CHC must host at least 2–3 specialist doctors (obstetrician/paediatrician/Ophthalmologist etc.). Designated referral facility based on the specialist present. Some CHCs may have facilities for Caesarean Births.	2–3 specialists, physicians, and SNMs
Health and Wellness Center: Primary Health Centers (PHCs)	4–6-bedded health facilities catering to a population of 25,000–30,000 [22]	Basic preventive and promotive healthcare with a strong focus on Maternal and Child Health programmes. Equipped to support normally progressing, low-risk pregnancies and births.	Physician supported by SNMs and ANMs
Sub-Center	No beds. Catering to 3000–5000 people	Primarily providing promotive and preventive health services. Vaccination, Family planning and health education	No medical doctor or specialist, no nurse-midwives or professional midwives. Functionalised by the ANM and Male multipurpose worker

We formed one technical advisory group representing government officers, development partners, UN agencies supporting the midwifery initiative, and midwives and obstetricians in each state. Two planning meetings with the advisory group in each state helped us select facilities that performed exceptionally well or poorly in integrating midwives and maximising diversity (including rural/urban, population characteristics, and service configuration/model of care). Based on the midwives’ availability, one MCH/DH and two CHCs were selected from each of the two states.

The key healthcare team personnel with whom the NPMs would need to integrate included the health facility superintendent (usually not an obstetrician) responsible for keeping the hospital operational and for liaising with district/state health departments; an obstetrician (with ultimate clinical authority within the hospital) and/or deputising medical officer; the SNMs who are trained to assist rather than lead care, working under the authority of the obstetrician/medical officer; and ANMs who are responsible for community-based health services and supporting SNMs in their tasks.

## Methods

### Study design and analysis

The research comprised a community-based and public health facility-based case study in two states in India using human-centred design. Ecological systems theory [23] and the Quality Maternal and Newborn Care (QMNC) Framework [23] were used to guide tool development, data collection, analysis and reporting processes. Qualitative framework analysis was used to analyse the interviews with NPMs, SNMs, ANMs, medical staff, hospital administrators, and policymakers.

Data generation and analyses for the community-level interviews were guided by Community-based Participatory Research (CBPR) principles. A human-centred design [24] was used to explore the women’s and community influencers’ perceptions of the quality of maternity care in general and as delivered by midwives and the acceptability of midwives as key providers of maternal and newborn care. Table 3 provides a brief description of the study’s methodologies.

**Table 3.** Description of methodologies and approaches used for data collection and analysis.

Method/Approach	Description
Ecological systems theory	The ecological systems theory employs a multi-level interactive approach to describe the interaction between and the interdependence of factors within and across all levels of health problems [23] and was used to form the key themes in this study. The ecological systems theory’s five levels of influencers are: 1) intrapersonal or individual factors (e.g. knowledge, perception, and beliefs of individuals); 2) interpersonal factors (e.g. the processes and influences caused by families, peers, groups etc.); 3) institutional or organisational factors (e.g. rules, regulations, policies); 4) community factors (e.g. social networks, standards, formal/informal social-cultural norms); and 5) public policy factors including local, state and national laws that regulate and enforce health practices [23]
QMNC Framework	The framework is based on 50 short—, medium-—, and long-term outcomes that could be improved by how and by whom care is provided [22]. The framework associated the care provided by educated, competent, licensed, and regulated midwives with improved maternal and neonatal morbidity and mortality [23]. The framework was used to explore the scope of midwifery practice as described by participants.
Framework Analyses	This pragmatic epistemological approach is well-suited for qualitative datasets generated from multi-disciplinary groups of participants [25]. The five steps of Framework Analysis were followed: 1) data familiarisation, 2) identifying a thematic framework, 3) indexing all study data against the framework, 4) charting to summarise the indexed data and 5) mapping and interpretation [25].
Human-centred Design	a practical methodology that keeps the end beneficiaries’ needs and demands in constant focus to promote better responses from service providers [24]

### Study participants

We purposively approached those identified as key stakeholders in administering and delivering maternal and child health services in the two states. From each state, we invited participation from those NPMs who had been deployed before 2019 (*n* = 13), SNMs (*n* = 8), obstetricians (or medical officers if the obstetrician was not available) (*n* = 8), and hospital administrators (*n* = 4) to represent their health facilities. Three state-level policymakers were also interviewed.

To represent community perspectives, pregnant women (*n* = 13) and new mothers (*n* = 13) assisted by midwives, other care providers or who had home births were interviewed. In addition, their family members and community influencers (including ANMs, ASHAs, members of the village governing body, police constable, and local shop owners popular in the community) participated (*n* = 14).

### Data generation

In-depth interview guides for all facility-based and community-based participants were prepared to ensure the NPMs’ role was explored within the context of other participants’ roles in maternal and child health care. Data were generated by experienced Research Assistants (RAs) – native speakers of Gujarati/Telugu and fluent in English – who had been trained for this purpose. The interviews/focus group discussions (FGDs) were conducted between September and December 2022 in a meeting room within the health facility (healthcare team members) or an identified community building (for community participants). Gujarati, Telugu, or English was used based on the participants’ language preference. The interviews lasted 40 minutes on average, and the FGDs 65 minutes.

## Data analysis

The five steps of Framework Analysis were followed across each case study (state-wise): Authors read, discussed and coded the transcripts to become familiar with the data. An analytical framework was developed using the Ecological model theory and the QMNC framework. Once all co-authors agreed on the framework, all data were indexed, and the mapping and interpretation exercise was performed. Each case study was analysed independently and then combined to generate common themes. The involvement of co-authors is presented in the ‘authors’ contribution’ section. This manuscript presents the findings using the collective data from the case studies in Gujarat and Telangana. Comparative findings between states are being developed as another manuscript.

## Findings

**Figure 1.**
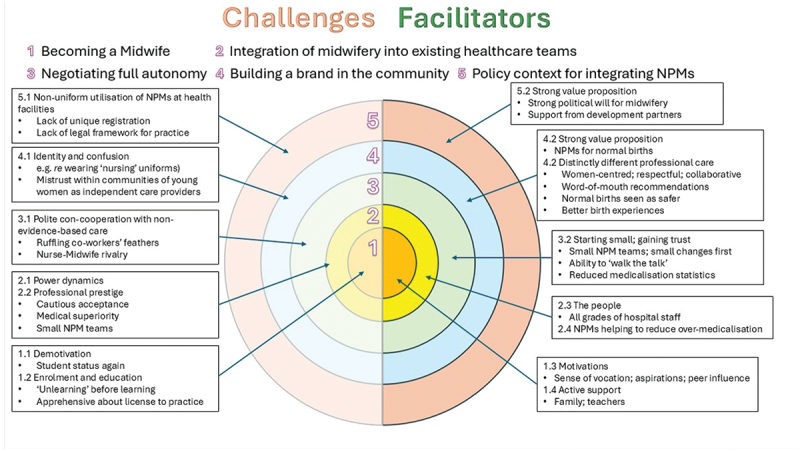
Facilitators and barriers to integrating midwifery in Indian public health systems.

Five themes were developed, each representing one level of the Ecological Systems Theory. Theme 1 *‘Becoming a midwife’*, corresponding to the intrapersonal level of the Ecological Systems Theory; Theme 2 *‘being accepted by the existing healthcare team’* corresponding to the intrapersonal level; Theme 3 ‘*Negotiating for full scope of autonomous midwifery practice’* corresponding to the systems level; Theme 4 ‘*Building social recognition of midwifery in the community’* corresponding to the community level; and, Theme 5 *‘The policy context for integration of NPMs’* corresponding to the policy level of the Ecological Systems Theory (Figure 1). The right side of the diagram presents the facilitators. In contrast, the left side of the figure presents the barriers to the acceptability and integration of midwifery and NPMs in the existing healthcare teams.

### Becoming a midwife

NPM participants described their ‘becoming a midwife’ journey in terms of their motivation to apply, their experiences of being selected and of undertaking the programme, and their experiences of becoming socialised into midwifery.

### Motivation to become a midwife

Motivation for NPMs arose from a sense of vocation, professional aspiration, and peer influence. A sense of vocation came from wanting to help mothers by providing quality midwifery care and helping to create positive birth experiences, driven for some by personal negative birth experiences. Others wanted to gain professional autonomy by becoming better educated and paid than SNMs or by observing the skill and competence of existing NPMs. NPM status meant a secure labour ward posting with the prospect of long-term future postings, rotating only within midwife-led care units (MLCUs). Some had applied on the advice of a supervising nurse-midwife or medical officer or because colleagues were applying.

I was a staff nurse in the obstetric ICU, where the senior NPM used to visit. She motivated me to enrol in the course. You know how popular (the NPM) is … … . (laughing). I was not aware of such a course … . Then we kept going to her for guidance about the admission process, etc. We learn a lot from her …. (NPM, Medical College Hospital, Gujarat)

### Experience of enrolment and NPM education

Newspapers in both states advertised the NPM courses. Applicants who passed a written test were personally interviewed. While the interviews in Gujarat seemed to explore applicants’ midwifery knowledge, the Telangana interviews focused on their reasons for wanting to be midwives.

Older NPMs experienced difficulties adjusting to students’ lifestyles, citing loss of seniority within the hospital hierarchy and the restrictions of hostel life, where a warden regulated them. The initial months of training saw some participants become demotivated, and some considered leaving.

I felt – instead of doing my job – suddenly I have to become a student again, which is very challenging. (NPM1, CHC, Gujarat)

… and you have to go back to the hostel at a particular time - the bus comes … they take attendance, it’s the (lack of) maturity level … I accepted, no doubt, we accept. Slowly the acceptance comes … (NPM2, CHC, Gujarat)

All, however, regained their motivation with support and counselling from family members and educators.

Most midwife participants felt surprised by the breadth and depth of the curriculum. They found both the classroom and clinical settings to be positive learning environments. Midwife participants respected their educators for their experience and use of diverse teaching-learning techniques. All students – medical, nursing, and midwives – learnt together. This promoted a sense of a **right to learn**, and motivated them to gain adequate clinical experience. This starkly contrasted participants’ earlier midwifery experiences during their nursing training, where they had often felt unwelcome in the labour wards.

We did not assist any birth as a student during our basic nursing and midwifery education …. I learnt on the job when I started working. But in NPM education we are legitimately allowed to and get a chance to learn to assist births independently as students. We can practice dedicatedly (In a safe environment) as we are assured of help from senior staff nurses as well as resident doctors (obstetric students). (NPM, CHC, Gujarat)

NPM programme coordinators in Telangana shared that they advocated with policymakers to make class sizes manageable (no more than 30 per class), encouraging adequate individual attention. They also protected clinical placements, ensuring adequate clinical experience – another stark contrast to their earlier nursing education experiences. Participants recalled getting support to practise hands-on midwifery care from resident doctors and existing NPMs. Night duty shifts offered more opportunities to practice independently.

### Socialisation into midwifery

Gujarat participants expressed a task-based understanding of midwifery. Since midwives in Gujarat were trained alongside obstetric resident physicians, they reported good clinical decision-making and an ability to recognise and manage first-level complications, ‘deep knowledge’, as expressed by a midwife from a CHC. By contrast, midwives from Telangana, mentored by international midwives, discussed core midwifery values such as women-centred respectful care and co-planning care with the mother. Telangana midwives recalled the challenge of having to ‘unlearn’ many things before they could adequately learn midwifery values.

I had to unlearn and learn evidence-based care. I realised that a pregnant woman is not a patient but a strong and healthy person who deserves our respect. I began to understand her choices and needs … This change in how we care for them is special. (NPM, Area Hospital, Telangana)

On graduation, participants from both states felt motivated, empowered, and confident, having imbibed midwifery’s core principles: women-centred respectful care and a trusting partnership between the woman and the midwife.

## Being accepted by the existing healthcare team

Participants described three aspects of this process of interpersonal integration: the *players*, the *power,* and the *professional prestige-related interplay*.

### The players

Participating midwives felt that preparing existing health teams (Superintendent, Administrator, Obstetrician/Physician, SNMs) for the midwives’ arrival was often inadequate. The non-midwife participants did not seem to understand the midwifery philosophy of evidence-based maternity care or how NPMs would integrate into the existing team. This lack of preparation disrupted team equilibrium and role confusion, leading to feelings of insecurity and even threat. Participants shared that conflicts were reduced when ‘sensitisation’ meetings were held, as in Telangana.

### The power

Participants reported differing levels of initial acceptability of midwives that seemed to be rooted in perceived changes to power dynamics. These levels were identified as *welcoming midwives*, cautiously accepting *midwives*, and *rejecting the need for midwives*.

They refused to accept me … said we do not want any NPM here. They are unaware of who an NPM is, even the head of the department. When we showed them our appointment order, they asked us, ‘Now, who is this NPM?’ They said they would not permit us to attend births independently. ‘I will count you as a staff nurse. You will do tasks as the staff nurse does … ’ So, I spent one month there and came to this hospital … (NPM, Medical College Hospital, Gujarat)

State-level policymakers and hospital superintendents responsible for reducing unnecessary clinical interventions in maternity care welcomed the midwives. They perceived midwives as credible normal birth ‘experts’ who would reduce non-evidence-based and costly interventions.

Before, the staff nurses’ demand for cerviprime [an induction gel], which is very costly, was very high … and then the women used to go for C section which is a meaningless waste of cerviprime …. After the midwives posting, it got reduced which became an excellent advantage for me … they are avoiding unnecessary scans also. (Superintendent, DH, Telangana)

Obstetricians at CHCs also welcomed the midwives, believing they could competently supervise all women in labour when the obstetrician was off duty.

NPMs are better than most senior staff nurses with experience; they have higher confidence, can deal with emergencies with clear and quick steps, have a better grasp, and follow instructions in one go……. NPMs do not fear or lose nerves during emergencies. They know what to do as they are dedicated. (Superintendent Urban CHC, Gujarat)

Midwives felt that their reception at multi-obstetrician health facilities was cautious. Being under scrutiny was initially tiring and demotivating, but they believed the best strategy was to let their work speak for itself. They also felt conscious that they were setting an example for future midwives.

In a few cases, midwives reported initial rejection, which could be openly or passively manifested. Examples included waiting outside the hospital authority’s office for hours or even days to meet and report officially for duty. On commencing, some were posted in non-maternity wards or instructed to work as an SNM. Younger midwives felt intimidated by obstetricians and senior SNMs and were reluctant to challenge non-evidence-based practices. To feel accepted, some midwives reverted to their old (pre-NPM) ways of practice.

In some instances, when informed of these issues of non-acceptance, state authorities exerted their power by issuing written orders safeguarding the midwives. Some even encouraged midwives to report untoward experiences directly to a state authority.

### Professional prestige-related interplay

The shifting power dynamics sometimes raised misgivings about different professional groups’ perceived importance and status. SNM participants unanimously reported being cooperative and even learning midwifery values from midwives. By contrast, the midwives felt that most of their professional conflicts were with SNMs, attributing this to apparent resentment at their better pay and their permanent attachment to the labour rooms. Midwives felt that SNMs initially guarded their territory, downplayed the midwives’ midwifery education, and either ignored or reversed clinical changes introduced by the midwives.

… but still, we had many quarrels with the SNMs because of the ‘this is your work, this is my work’ attitude. Then slowly they too started accepting, but it still became shaky … sometimes (laughs), some quarrels break out, we had to go to (superintendent) to sort it out …. (NPM, CHC, Gujarat)

While many non-midwife participants expressed how midwives had improved care quality, a few medical officers/obstetricians felt that the midwives had not introduced any noteworthy changes.

NPMs will do what we used to do. They have to learn from us; we don’t (need to learn from them). Because we are doing much more than the midwives. In high-risk cases, they still need to learn the protocol and how to implement it. (Obstetrician, CHC, Telangana)

The hospital superintendents remembered having to adopt early conflict resolution by convincing different groups how the midwives would benefit all.

There was no good coordination between obstetricians and midwives because of ego problems. Now, I have convinced the obstetricians that midwives came here to reduce/share your burden of work; then they got convinced, and now they become friends. (Superintendent, DH, Telangana)

## Negotiating for full scope of autonomous midwifery practice

Midwives reported that their scope of midwifery practice ranged from the full scope in some facilities to work on a par with SNMs in others. The QMNC framework used to map the scope of practice currently performed by the midwives showed that the scope was focused mainly on intrapartum care and immediate postpartum and newborn care in line with the scope of practice document by the Indian Nursing Council [26], instead of the global standards. Only a few health facilities allowed the midwives to set up their own ANC clinics. Involvement in postpartum and newborn care beyond a few hours post-birth seemed missing. Intrapartum care by the midwives was under the direct supervision of the obstetricians and did not seem autonomous yet.

Multiple strategies were adopted to negotiate their full scope of practice. Midwives sometimes negotiated individually within their health facility without requesting government authority support. More commonly, the negotiation involved support and mentoring from their educators or a government authority. The common strategies cited by the midwives, and their mentors/supporting authorities were:

*Walking the talk*: Midwives recalled being introduced to the existing healthcare teams as ‘experts in normal birth’, and their ability to successfully support women in labour and birth (normal or with complications) was the most effective way of demonstrating they could be trusted to practice autonomously.

We faced challenges in performing to our full scope of practice during the initial year of posting; later, we negotiated with the superintendent and doctors. We requested doctors to observe our work, which helped gain their trust. Even now, we present monthly data to doctors and discuss with them the case studies that were challenging to us. Now, the doctors refer the low-risk pregnancies to us. (NPM, DH, Telangana)

*Starting small*: Midwives and their mentors/supporting authorities recalled challenges in practising midwifery in higher-level hospitals with high workloads. Acute staffing shortages predisposed to a fast-paced, task-oriented service delivery that left little scope to establish ‘midwifery’ processes within the medical model of care. The policymakers responded by re-posting midwives to CHCs, which served two purposes: it provided a competent maternity care provider to remote CHCs or CHCs without an obstetrician, and it offered a work environment that gave the midwives the initial breathing space to establish their practice in line with the midwifery model of care.

Their mentors counselled midwives to start with minor changes in practices (e.g. mobilising women in early labour, maintaining upright positions) before trying to tackle more significant changes, such as challenging harmful practices (e.g. applying fundal pressure, liberal use of episiotomies and oxytocics).

*Politely resisting non-evidence-based care: This* was taught to the midwives during their training. The midwives knew they were the pioneers of a new profession in India and that professionalism was always crucial in developing midwifery’s credibility within their hospitals. When witnessing non-evidence-based care, they often politely declined to participate. Sometimes, this strategy successfully got their colleagues to reflect on the need for the intervention. However, if their colleagues carried on regardless, the midwives often felt frustrated, as if they had failed.

All our midwives refuse to participate in any harmful practice … you have situations where a colleague takes scissors and just cuts (i.e. performs episiotomy) and this has been quite difficult for some of our trainees, even seniors have been reduced to tears because they’re now beginning to understand what obstetric violence is. (NPM educational programme coordinator, Telangana)

“… But we’ve promised that they will never argue in the delivery room in front of the mother, no matter how angry they become. We will have the discussions outside later. Because the last thing we want is the obstetric fraternity to feel (professionally upstaged). (Senior Policy Maker, state anonymised)

Participants identified several factors that influenced the midwives’ negotiation abilities, such as age, experience and personality of the midwife. Older midwives seemed more assertive in negotiating their scope of practice with other professionals. Their professional seniority seemed to gain them better professional respect. By contrast, younger midwives often experienced being labelled ‘inexperienced, thus less competent than older midwives’. The terms ‘old’ and ‘young’ referred primarily to age but also to perceived experience. All participant groups felt that ‘Older’ connoted greater experience. The longer the midwives served at one specific facility, the better they became at articulating and demonstrating the midwifery philosophy in hands-on care and building a stronger case among their colleagues for autonomous midwifery practice.

## Building social recognition of midwifery in the community

The midwives perceived that women and families commonly associated institutional birth with an obstetrician-led care model. Through mentoring or self-experience, the midwives had learnt that being accepted by and in demand within the community was essential to establishing midwifery as an independent profession.

This social recognition of midwifery in the community included ‘*distinguishing the midwifery model from the medical model of care’*, ‘*valuing NPMs as ‘experts in normal birth’*, and *‘harnessing word-of-mouth’* within the community.

*Distinguishing the midwifery -model from the medical- model of care*: The midwives recalled working hard to ensure that birth experiences were women-centred, respectful and inclusive of women in decision-making. The interviewed women reported needing to learn what to expect during birth, going beyond the physiological changes, and more regarding hopes and fears. This information was generally not part of the care package until they met the midwives. Interviewed women noted midwives’ efforts to establish professional relationships and provide continuity of care, which seemed to build midwifery’s social recognition within the community.

When I was having back pains, the NPM massaged my back and gave me hot water to drink. To make it more comfortable, she drank hot water alongside me to give me confidence. She motivated me, made me believe I could have a normal birth. (Primigravida, Rural, Telangana)

Doctors are there, but they are not so approachable. … reaching out to NPMs is more accessible due to their friendly nature. (Multigravida, Rural, Gujarat)

*Valuing NPMs*: Participating care providers and hospital stakeholders commonly referred to the midwives as ‘experts in normal birth’. Local postpartum women recalled that the midwives’ calm assurances and information-sharing practices alleviated their anxieties in both pregnancy and labour. This also encouraged them to choose normal births rather than demanding augmentation/intervention during labour.

The private doctor says that this is the third child, so a C-section will be done. Such suggestions depress one so. But here (public facility), my own Brother-in-Law is the medical officer, and it is an NPM-led facility, and they said I will have a normal birth. That is why I have been seeking treatment here with the NPMs. (Multigravida, Urban, Gujarat)

I gave birth two days after the due date, so I was worried and asked the NPM if I should take any injections or medication. Instead, the midwife suggested that I exercise and walk to have a normal delivery. (Primigravida, Urban, Telangana)

Women’s trust in midwives grew when they could experience normal birth, which most women participants wanted. Women who had previously given birth with care from any other healthcare provider deemed their birth experience with midwives to be better.

During my second pregnancy, they (care providers) kept me lying down and … ignored me for nearly two hours after the birth. I faced many problems due to this neglect. That is why I did not want to return to that facility, as it still did not have an NPM. (Multigravida, Rural, Telangana)

The support of policymakers, who saw them as a sustainable solution to reducing the over-medicalization of childbirth, also established the NPMs as the experts on normal births.

…. the C-section rate and our Maternal Mortality Ratio in the tribal and remote areas is very high. I was looking for a solution, and that is how I came across this solution of nurses further educated as midwives. It was a pilot which we started with XXX Agency, and we started with a batch of 30. When we started it, I don’t think we understood the transformational change it would make. (Policy Maker, Telangana)

*Harnessing word-of-mouth*: The women the midwives attended recalled encouraging friends and families to seek midwives. The midwives’ interactions with women and family members also helped increase their community visibility. The family members who initially perceived the midwives as ‘too young and inexperienced in handling independent care’ witnessed their work with women first-hand and gradually changed their opinion.

Midwife gives good counselling on healthy food, what to eat, what exercises are needed for normal delivery - … I am happy that NPMs do normal delivery and make women comfortable and confident … and this is what I have been telling others. (Husband of a primigravida, Urban, Telangana)

My relatives and friends who gave birth recently told us about NPMs and the quality services they provide. They also told us about the psychological support they provide before and during the delivery time in the maternity room. (Telangana)

Every week, we are getting excellent feedback. … Up to now they are not aware (of the term midwife) but occasionally I can see the word ‘midwife’ coming up in feedback forms so I can say that the word is going into the public slowly. (Medical Superintendent, District Hospital, Telangana)

One key challenge in establishing the identity of midwives was verbalised by the Gujarat NPMs, whose uniforms were indistinguishable from the SNMs’. By contrast, Telangana NPMs had a distinctive uniform which helped them establish their identity.

## The policy context for integration of NPMs

Strong political backing for midwifery was reported to be the most significant factor in integrating it within existing health systems. Support from development partners and the Government of India in introducing midwifery as an independent profession complemented the political will. However, despite continued overall policy-level support, some policy-related challenges remained.

### Lack of unique registration

At the time of our study, the midwives had no unique registration to set them apart from the SNMs. Midwives continued to work with their RN RM dual registration, making it difficult for others to distinguish them from SNMs and diluting midwives’ autonomy.

### Lack of an operational, legal framework of practice

At the time of our study, no legal framework for midwifery practice existed, which meant that obstetricians remained legally responsible for the outcomes of all women and neonates in their health facilities. Again, this reduced the impact of any advocacy for autonomous midwifery practice. Some policymakers seemed to question the need for the 18-month-long programme to prepare midwives, revealing they probably still considered midwives to be specialised nurses.

I don’t know if this 18-month (education to become NPMs) is really needed … It could be a six-month training and possibly six months on the job assisting somebody and then slowly (becoming independent) … Many of them (as SNMs) are practising all of this already. (Policymaker, state anonymised)

### Non-uniform utilisation of midwives at the institutional levels

The participants’ experiences in this study demonstrate several institutional-level arrangements and ground-level negotiations that have introduced a non-uniform policy implementation.

Participating midwives indicated that they still juggled midwifery practice with nursing responsibilities delegated to them; furthermore, non-midwife participants felt that utilising midwives in non-midwifery areas was acceptable in understaffed health facilities.

The strategy of posting a group of midwives to a facility was raised. A single midwife within a health facility often found it more challenging to negotiate the full scope of practice than when a small group of three or more (team) were posted to the same facility. Team posting made covering different shifts feasible and predisposed to better continuity of care for women in labour; demonstrating the benefits of good midwifery practice helped sustain the midwives. However, this strategy inherently reduced a midwife’s ability to choose where she would work.

Initially, the midwives were given posting as per their choice, resulting in some facilities having one or two midwives, which did not serve the purpose of continuity of care. So within four months, the midwives were counselled on the importance of working in clusters, that is, a minimum of four to five and were assured that once the second batch of NPMs completed their training, the pilot batch could go back to the facilities as per their choice, this arrangement was accepted by the pilot batch of midwives and was posted in clusters based on the workload of the facility. (NPM programme coordinator, Telangana)

## Discussion

There is high-level evidence that having midwives trained to international standards can significantly reduce maternal and perinatal mortality, as well as improve a wide range of other outcomes, including preterm birth rates [1]. Our study confirmed that introducing professional midwives into existing health teams, however, is a complex intervention and not without its challenges. Our findings reveal that the midwife participants had to unlearn and relearn a lot to become socialised into midwifery, and often experienced apprehension, uncertainty, and demotivation when they truly understood the significant professional change they had committed to, by joining the training. These accounts reveal an opportunity to improve the counselling and mentoring provided to the midwives during their training to help their transition from nurse-midwives to professional midwives. Becoming a midwife has been described as a life-altering experience requiring support from preceptors, educators, and mentors of midwifery students in other qualitative studies as well [27,28]. A scoping review of published literature from Canada, the United Kingdom, the Netherlands, Australia, and New Zealand by Gray et al. [29] showed that midwifery students found their transition to independent professional midwives rewarding but stressful, as it took time to gain confidence. A recent review [30] described three types of mentoring processes: student-to-student (peer), midwife-to-student, and midwife-to-new graduate midwife. All three could be tried in the Indian context to identify how theory can be tailored to ensure a culture of mentoring and support among midwives that benefits India and other countries transitioning to a midwifery model of care.

Our study showed that all participating midwives struggled to establish autonomy and the midwifery model of care with the full scope of midwifery practice in integrated teams. The obstetricians, physicians, SNMs and ANMs who took part in our study generally perceived midwives as ‘experts of normal birth’; however, these participants also expressed some pre-judgment and feelings of rivalry. This finding resonates with findings from other Southeast Asian countries that have recently transitioned to midwifery models of care [31,32] and from both HICs [33] and other LMICs [34] having a long history of professional midwifery [35]. This finding demonstrates a need to develop sensitisation training for existing members of maternity healthcare teams to prepare them for the introduction of midwives. The sensitisation is recommended by the Indian Nursing Council’s Guidance note [36] as well. This study reveals that the sensitisation training kit, which highlights how the midwives’ presence benefits other health team members, could improve the buy-in of the different healthcare team members and strengthen collaborative care to ensure universal health coverage and quality care to all women, their neonates and families. For example, promoting midwives as the experts of normal births without acknowledging the contribution of SNMs in providing maternity care until the introduction of midwives could sow seeds of professional conflict that can easily be minimised through respectful collaboration. Obstetricians’ sensitisation is equally required to accept midwives as an autonomous cadre; this study and studies from elsewhere [35,37] have revealed that professional midwives may be perceived as ‘less’ of a profession or as ‘specialised nurses’ [38]. Such rivalries and identity confusion could undermine the establishment and sustainability of midwifery as a new cadre of professionals. Systematic sensitisation could be a pragmatic, sustainable, and cost-effective mitigation strategy.

Our study shows that strong word-of-mouth-driven social recognition was building in favour of midwives. This led to a consistently growing awareness of midwives in communities. This finding resonates with evidence from another qualitative study [39]. Strong community demand could become an impactful and cost-effective strategy for promoting the integration of midwives into the health system.

This study provides insight into the initial facilitators and barriers to the acceptability and integration of midwives, which is a necessary component of transitioning to full implementation of midwifery models of care in which midwives work autonomously while collaborating with other members of the multi-disciplinary team. Our work provides a foundation for planning further research and interventions supporting this process.

### Strengths and limitations

This study aimed to gather primary evidence on facilitators and barriers driving the acceptability and integration of midwifery and NPMs in existing public healthcare systems in India. The study rigorously followed case study and framework analysis methodologies, used by a group of experienced qualitative researchers. The study had multi-disciplinary participation, representing and triangulating the findings from health systems and community, the first study to do so in India to the best of our knowledge. Having researchers with expertise in co-designing and community participatory research further strengthened our research processes.

One co-author’s Foundation has pioneered the NPM programme in Telangana. Therefore, their positionality in the study may have created some bias. As a mitigation strategy, the qualitative interviews were conducted by two research associates who were not affiliated with the foundation. The findings come from only two Indian states; therefore, other facilitators and barriers may exist as the roll-out is extended to other states.

## Conclusion

This study showed that the integration of NPMs into the existing health system poses multi-faceted challenges and could be improved by: 1) providing counselling to aspiring NPMs during their 18-month NPM education to overcome their misgivings and to facilitate their journey towards becoming a midwife; 2) developing customised sensitisation training to improve the existing maternity care health team’s awareness about midwives and their role within the health team; 3) by fostering an enabling environment that supported the NPMs without downplaying the important roles played by other healthcare providers such as obstetricians and nurse-midwives and actively promoted a collaborative care model; and 4) by helping the NPMs in building a strong social recognition at the community level by creating more visibility and value for the new cadre. Having a higher pay scale for midwives, sensitisation to create a separate image of midwifery from nursing and developing midwifery-specific monitoring indicators and reporting systems could improve the midwives’ professional visibility.

## Data Availability

Authors declare that the anonymised data can be accessed at FRHS archives upon reasonable requests.
